# Targeting ApoA5‐Associated Hypertriglyceridemia to Ameliorate Acute Pancreatitis: Insights From a Knockout Hamster Model

**DOI:** 10.1002/mco2.70555

**Published:** 2025-12-15

**Authors:** Sijing Shi, Kaikai Lu, Yijun Tao, Yue Zhang, Ling Zhang, George Liu, Wei Huang, Yuhui Wang, Xunde Xian

**Affiliations:** ^1^ Institute of Cardiovascular Sciences State Key Laboratory of Vascular Homeostasis and Remodeling School of Basic Medical Sciences Peking University Beijing China; ^2^ Department of Health Management The First Affiliated Hospital Jiangxi Medical College Nanchang University Nanchang China

1

Dear Editor.

While gallstones and alcohol are leading causes of acute pancreatitis (AP), emerging evidence highlights a significant role for genetic and metabolic factors, particularly in recurrent AP (RAP) [1]. Among them, severe hypertriglyceridemia (HTG) is a key risk factor, though its mechanistic link to AP remains unclear.

Previous studies have demonstrated that several mutations in human genes regulating triglyceride metabolism, in which *APOA5* is one of the most common genetic abnormalities in RAP patients [2]. However, the relationship between ApoA5 and HTG‐AP has not been well investigated in experimental animals. Recently, we developed *Apoa5* knockout (*Apoa5^−/−^*) golden hamster model that reproducibly exhibited severe HTG, closely resembling human familial chylomicronemia syndrome [3]. Importantly, we observed that the activation of UCP1 in brown adipose tissue (BAT) by cold exposure or the β3‐adrenergic receptor agonist CL316243 administration could significantly correct severe HTG and hepatic steatosis in *Apoa5^−/−^* hamsters [3]. The incidence of AP serves as a key metric for evaluating the therapeutic effectiveness of triglyceride‐lowering agents in clinical trials; however, whether correction of severe HTG can improve the outcome of HTG‐associated RAP has not been investigated in our hamster model yet. In the present study, it is rational for us to further investigate the relationship between BAT activation and pancreatic pathology in the context of ApoA5 deficiency.

In agreement with the previous findings [4], AP was established in this hamster model by intraperitoneal caerulein injection (Figure 1A and Supporting information). Plasma triglyceride and amylase levels in *Apoa5^−/−^* hamsters were significantly increased compared with WT controls. Histological examination revealed markedly aggravated pancreatic injury in *Apoa5^−/−^* animals. Consistently, histopathological scores were significantly increased compared with WT controls. Myeloperoxidase expression and apoptotic bodies were increased in pancreas of *Apoa5^−/−^* hamsters, indicating enhanced local inflammation and cell death. Moreover, mRNA expression profiling demonstrated an upregulation in proinflammatory cytokines (*Tnfα, Cd68*), the apoptotic marker *Bax*, and a downregulation in the anti‐inflammatory cytokine *Il10* (Figure 1B).

**FIGURE 1:**
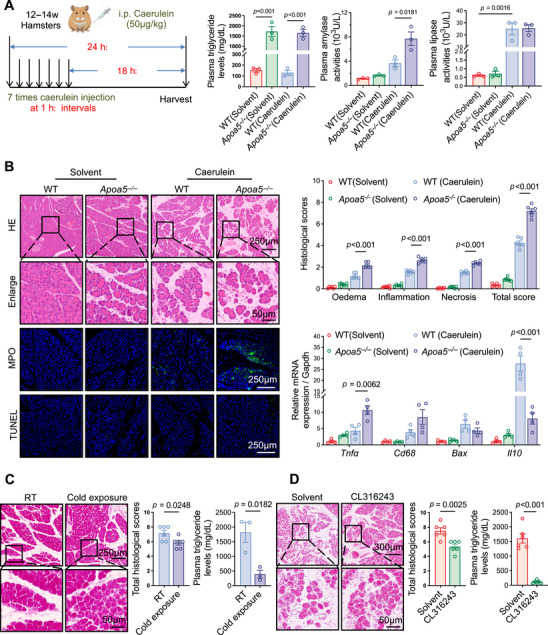
*Apoa5* deficiency worsens acute pancreatitis (AP) via chylomicron accumulation, which is ameliorated by lipid‐lowering interventions. (A) The schemic representation of hamster model with AP and plasma triglyceride, amylase, lipase levels from wild type (WT), and *Apoa5^−/−^* hamsters with or without caerulein treatment. (B) Representative images of H&E, myeloperoxidase (MPO), and TUNEL staining in pancreatic sections from the indicated animals (left). Scale bar, 250 and 50 µm. Histological injury scores from the indicated animals, mRNA expression of *tumor necrosis factor α (Tnfα)*, *cluster of differentiation 68* (*Cd68), BCL2‐associated X (Bax)*, and *interleukin 10 (Il10)* in pancreatic tissues from the indicated animals with or without caerulein treatment (right). (C) Pancreatic H&E staining in *Apoa5^−/−^* hamsters under cold exposure or room temperature (RT) condition. Scale bar, 250 and 50 µm (left). Histological injury scores and plasma triglyceride levels in the indicated animals (right). (D) Histological analysis in *Apoa5^−/−^* hamsters with or without CL316243 treatment (left). Scale bar, 300 and 50 µm. Histological injury scores and plasma triglyceride from the indicated animals (right). All data presented as mean ± SEM. Student's *t*‐test or two‐way ANOVA used for statistical analysis.

To our knowledge, it is very difficult to treat familial HTG. However, we found unexpectedly that acting through stimulation of BAT metabolism significantly reduced plasma triglyceride levels in *Apoa5^−/−^* hamsters [3]. Herein, we tested whether this therapeutic avenues could improve pancreatic outcomes in *Apoa5* ^−/−^ hamsters with AP. Cold exposure at 4°C for 16 h significantly lowered plasma triglycerides (Figure 1C) and then ameliorated pancreatic pathological injury. Similarly, treatment with CL316243 to activate BAT led to a substantial reduction in plasma triglyceride levels, as well as an improvement of pancreatic histopathology (Figure 1D).

In summary, our study establishes *Apoa5* deficiency as a potent genetic driver of HTG‐AP through chylomicron accumulation in circulation. Importantly, we show that pharmacologic or environmental activation of BAT‐associated lipid metabolism can reverse the pathological features of HTG‐AP, offering a clinically translatable, nonviral, and low‐risk strategy. Our findings underscore the importance of genetic screening and triglyceride monitoring in RAP patients, supporting the development of metabolism‐targeted therapies as a novel approach to treat HTG‐AP. However, it should be noted that the species differences in ApoA5 biology, BAT activity, and dietary influences cannot be excluded when translating the findings reported in hamster model to humans, which need to be further studied in future.

## Author Contributions

Study concept and design: Yuhui Wang and Xunde Xian. Experiments: Sijing Shi, Kaikai Lu, Yijun Tao, Ling Zhang, and Yue Zhang. Acquisition, analysis, or interpretation of data: Sijing Shi and Kaikai Lu. Drafting of the manuscript: Sijing Shi, Kaikai Lu, Geoger Liu, Wei Huang, Yuhui Wang, and Xunde Xian. Critical revision of the manuscript for important intellectual content: Yuhui Wang and Xunde Xian. Obtained funding: Yue Zhang, Yuhui Wang, and Xunde Xian. Final approval of manuscript as submitted: Xunde Xian. Guarantor of the article: Xunde Xian. All authors have read and approved the final manuscript.

## Funding Information

This work was supported by the National Natural Science Foundation of China (NSFC) 82270479 and HY2021‐1; Peking University Medicine plus X Pilot Program‐Platform Construction Project 2024YXXLHPT010 to X.X.; the Beijing Natural Science Foundation Z230017 to Y.W.; the Jiangxi Provincial Natural Science Foundation 20212BAB216022 to Y.Z.

## Ethics Statement

All experiments were approved by the Laboratory Animal Ethics Committee of Peking University (Approval No. LA2022147).

## Conflicts of Interest

The authors declare no conflicts of interest.

## Supporting information




**Supporting File 1**: mco270555‐sup‐0001‐SuppMat.docx

## Data Availability

The data generated are available from the corresponding author upon reasonable request.
