# Supplementary material for: Targeting ApoA5‐Associated Hypertriglyceridemia to Ameliorate Acute Pancreatitis: Insights From a Knockout Hamster Model
Source: MedComm (2020). 2025 Dec 15;6(12):e70555. doi: 10.1002/mco2.70555 (PMC12706169; doi:10.1002/mco2.70555)
Supplement: Supplementary file 1 — Supporting File 1: mco270555‐sup‐0001‐SuppMat.docx [file MCO2-6-e70555-s001.docx]

Supplementary Documants

**Targeting ApoA5-associated hypertriglyceridemia to ameliorate acute pancreatitis: insights from a knockout hamster model**

Sijing Shi^1,#^, Kaikai Lu^1,#^, Yijun Tao^1^, Yue Zhang^2^, Ling Zhang^1^, George Liu^1^, Wei Huang^1^, Yuhui Wang^1,*^，Xunde Xian^1*^

^1^Institute of Cardiovascular Sciences, State Key Laboratory of Vascular Homeostasis and Remodeling, School of Basic Medical Sciences, Peking University, Beijing, China.

^2^Department of Health Management, The First Affiliated Hospital, Jiangxi Medical College, Nanchang University, Nanchang, China.

^#^Sijing Shi and Kaikai Lu contributed equally to this work.

*Correspondence to: Xunde Xian, Email: [xianxunde@bjmu.edu.cn](mailto:mailto:xianxunde@bjmu.edu.cn); Yuhui Wang, Email: wangyuhui2009@bjmu.edu.cn.

**Materials and Methods**

***1. Animal***

The source of syrian golden hamsters used in our lab is originally from Vital River Laboratory (Beijing, China). *Apoa5^-/-^* hamsters were generated in our lab by CRISPR/Cas9 gene editing system previously^1^. For all experiments, all animals were housed in a stable temperature and humidity environment under a 14-hour light/10-hour dark cycle at 24 °C, with chow diet (20% protein and 4% fat; Beijing Ke’ao Company, Beijing, China) and water *ad libitum*. All experiments were performed under the principle of experimental animal care (NIH publication No. 85Y23, revised 1996) and were approved by the Laboratory Animal Ethics Committee of Peking University (Approval No. LA2022147).

***2. Caerulein-induced acute pancreatitis model***

WT and *Apoa5^-/-^* hamsters aged 12-14 weeks were randomly assigned to solvent control or caerulein treatment groups (n=6/group). Animals received intraperitoneal injections of caerulein (50 μg/kg; AS-24252, AnaSpec, USA) once per hour for a total of seven injections.

***3. Cold exposure***

Male *Apoa5^-/-^* hamsters (12-14 weeks old) were randomly divided into two groups: one group was maintained at room temperature, while the other was subjected to cold exposure (4℃) for 16 hours. Both groups were administered intraperitoneal injections of caerulein once per hour for seven doses. Animals were sacrificed 24 hours post-induction for sample collection.

***4. Treatment of CL316243***

Male *Apoa5^-/-^* hamsters (12-14 weeks old) were randomly allocated into two groups and subcutaneously injected in the dorsal neck region with either PBS or CL316243 (600 μg/kg/day; AB144605, abcam, USA) for seven consecutive days. Both groups were administered intraperitoneal injections of caerulein once per hour for seven doses. At the end of each experimental protocol, animals were euthanized. Blood and pancreas tissues were harvested for further analysis

***5. Biochemical measurements***

We randomly collected three plasma samples from each group at 24 hour after caerulein induction for biochemical analysis. Plasma triglyceride levels were measured using a commercial enzymetic assay kit (Biosino Bio Technology & Science, Beijing, China) as the manual. In brief, 5 µL of plasma and 200 µL of enzyme reagent were mixed in a 96-well plate and was incubated at 37℃ for 10 min.Then the plate was read at 492 nm using a microplate reader (CLARIOstar, BMG LABTECH, Germany). Triglyceride concentrations were calculated based on the standard curve. Amylase and lipase activities were tested from plasma collected at 24 h after the first caerulein injection. Then measured by the Catalyst Dx Chemistry Analyzer (IDEXX Laboratories, Inc., Westbrook, ME) using the DRI-CHEM method. Plasma samples for amylase and lipase tests were preprocessed by ultracentrifugation at 25,000 rpm for 30 min at 4℃ to avoid lipemia interference.

***6. Histopathology***

Tissue samples were collected at 24 hour after caerulien induction. Pancreatic tissues were fixed in 10% neutral-buffered formalin and subsequently embedded in paraffin. Tissue sections were stained with haematoxylin and eosin (H&E) to evaluate histopathological changes. Histological scoring of pancreatic injury was performed according to previously established criteria^2^. Neutrophil infiltration in the pancreas was assessed by myeloperoxidase (MPO) immunofluorescence staining(ab9535, abcam, USA). Apoptotic cell death was evaluated using TUNEL staining (40308ES50, Yeasen, China; BZ-X800, Keyence, USA).

***7. Real-Time Quantitative PCR (RT-qPCR) Analysis***

Total RNA was extracted from pancreat tissues using TRIzol reagent (NEP019-2, Dingguo; China) according to the manufacturer’s instructions. A total of 5 μg RNA was reverse transcribed into complementary DNA (cDNA) using the RevertAid Reverse Transcription Kit (Thermo Scientific, M1635, USA). Quantitative real-time PCR (RT-qPCR) was performed using SYBR Green (AQ132-23, TransGen, China) to assess mRNA expression levels. Relative gene expression was normalized to Gapdh as an internal control.

**REFERENCES**

1. Guo J, Miao G, Zhang W, et al. Depletion of ApoA5 aggravates spontaneous and diet-induced nonalcoholic fatty liver disease by reducing hepatic NR1D1 in hamsters. *Theranostics*. 2024;14(5):2036-2057.

2. Wang Y, Kayoumu A, Lu G, et al. Experimental Models in Syrian Golden Hamster Replicate Human Acute Pancreatitis. *Sci Rep*. 2016;6:28014.
